# Conceptualisation of severe and enduring anorexia nervosa: a qualitative meta-synthesis

**DOI:** 10.1186/s12888-023-05098-9

**Published:** 2023-08-18

**Authors:** Laura Kiely, Janet Conti, Phillipa Hay

**Affiliations:** 1 School of Medicine, Translational Health Research Institute, Western Sydney University, Sydney, NSW Australia; 2 School of Psychology, Translational Health Research Institute, Western Sydney University, Sydney, NSW Australia; 3 School of Medicine, Translational Health Research Institute, Western Sydney University. Mental Health Services, Camden and Campbelltown Hospitals, SWSLHD, Campbeltown, NSW Australia

**Keywords:** Severe and enduring anorexia nervosa, Qualitative meta-ethnography, Meta-synthesis, Inter-psychic, Intra-psychic, Lived experience, Recovery, Treatment

## Abstract

**Background:**

Severe and enduring anorexia nervosa (SE-AN) is amongst the most impairing of all mental illnesses. Collective uncertainties about SE-AN nosology impacts treatment refinement. Qualitative research, particularly lived experience literature, can contribute to a process of revision and enrichment of understanding the SE-AN experience and further develop treatment interventions. Poor outcomes to date, as evidenced in clinical trials and mortality for people with SE-AN (1 in 20) demonstrate the need for research that informs conceptualisations and novel treatment directions. This interpretative, meta-ethnographic meta-synthesis aimed to bridge this gap.

**Methods:**

A systematic search for qualitative studies that explored the AN experiences of people with a duration of greater than 3 years was undertaken. These studies included those that encompassed phenomenology, treatment experiences and recovery.

**Results:**

36 papers, comprising 382 voices of SE-AN experiences informed the meta-ethnographic findings. Four higher order constructs were generated through a synthesis of themes and participant extracts cited in the extracted papers: (1) Vulnerable sense of self (2) Intra-psychic processes (3) Global impoverishment (4) Inter-psychic temporal processes. Running across these meta-themes were three cross cutting themes (i) Treatment: help versus harm, (ii) Shifts in control (iii) Hope versus hopelessness. These meta-themes were integrated into conceptualisations of SE-AN that was experienced as a recursive process of existential self-in-relation to other and the anorexia nervosa trap.

**Conclusions:**

The alternative conceptualisation of SE-AN proposed in this paper poses a challenge to current conceptualisations of AN and calls for treatments to engage with the complex intra and inter-psychic processes of the SE-AN, more fully. In doing so, clinicians and researchers are asked to continue to be bold in testing novel ideas that may challenge our own rigidity and attachment to dominant paradigms to best serve the individual person with SE-AN. The ‘global impoverishment of self’, found in this synthesis of AN experiences, should inform proposed diagnostic criteria for SE-AN.

**Supplementary Information:**

The online version contains supplementary material available at 10.1186/s12888-023-05098-9.

## Background

Severe and enduring eating disorders (SE-ED; [1]), are amongst the most impairing (physical, emotional, fiscal and social) of all mental illnesses [2]. Around 20 percent of individuals who experience anorexia nervosa (AN) have a protracted illness course [3, 4]. Severe and Enduring Anorexia Nervosa (SE-AN), is the current nosology for these long standing cases. Despite SE-AN being described in the early 1980’s [5], the defining features are incomplete [6, 7], with little consensus about optimal treatments. Although SE-AN has been acknowledged within clinical practice guidelines in the last decade [8], unresolved questions persist. These include (a) relationship between SE-AN and other SE-ED, (b) how severity is defined outside of illness duration and medical variables e.g. body weight (c) treatment resistance versus treatment inadequacy, and (d) defining best practice SE-AN treatments [9–13].

Manualized psychological therapies are the first line in care for most people with AN but to our knowledge only one group has developed a treatment intervention for SE-AN and tested this with a randomized controlled trial [14]. Notably, in the trial, treatment goals were modified in accordance with a broader conceptualisation of recovery with the treatment focus on quality of life rather than reduction of ED symptoms. This aligned with prior lived experience research where recovery has been argued to be more than symptom reduction or remission [15]. Duration of AN was the only delineating factor for participants, where other putative features of SE-AN have since been proposed. There is a paucity of research on AN intervention with a broader focus and scope to be flexibly tailored to the individual’s unique needs and preferences [16, 17]. Given the collective uncertainties about the “right” treatment for the “right” person at the “right” time and the poor outcomes to date [18], there is a clear need for research to comprehensively inform conceptualisations of SE-AN and innovate treatment interventions.

Complex questions demand complex forms of evidence [19] and in (re)configuring understandings of SE-AN and its treatment, all forms of evidence potentially offer a vital contribution. Qualitative research, particularly lived experience literature, can contribute to a process of revision and enrichment of understanding rather than being limited to the verification of earlier conclusions or theories. A synthesis of existing qualitative research therefore has scope to generate: (a) rich understandings of SE-AN from the perspective of the experiencing person; (b) new ideas for testing; and (c) contributions to innovations in treatment. The latter is especially important in the current context where there is a narrow selection of evidence-based treatments for AN, which may themselves be iatrogenic [20].

To our knowledge, there are seven meta-syntheses of qualitative AN literature with differing foci, including recovery [21–23], phenomenology of AN [24, 25], and treatment experiences [26, 27] and three additional, encompassing all eating disorders, including AN [15, 28, 29]. However, none of these systematic reviews have focused on people who experience SE-AN and the perspective of longstanding AN in those who have had treatment has yet to be delineated. Thus, this meta-synthesis aims to contribute to the body of literature seeking to understand and conceptualize AN from the perspective of those who have experienced a protracted course, despite treatment.

### Review questions

This meta-synthesis of qualitative studies seeks to deepen understandings of the experiences, treatment, and recovery of people with SE-AN with the aim to generate a person-centred conceptualisation to inform current and future treatment interventions. Accordingly, the review questions were: (1) How do people experience SE-AN? (2) What are people’s perceived treatment needs and do treatments meet these? and, (3) what role does treatment play in recovery, and does it interact with illness persistence from the perspective of those who experience AN?

## Methods

**Fig. 1:**
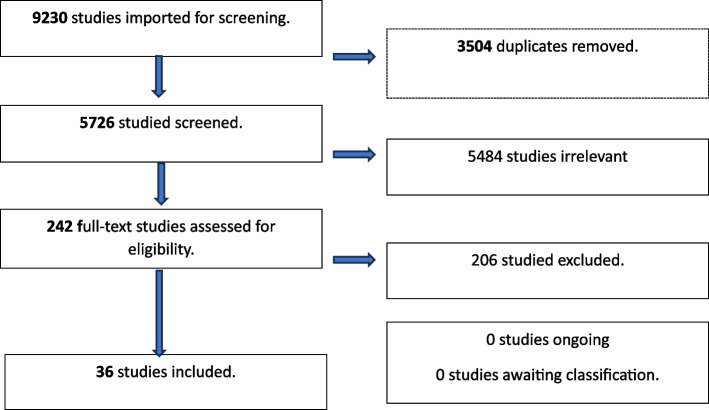
Covidence PRISMA flow chart of search strategy

Qualitative papers that focused on SE-AN were identified systematically (Fig. 1 – Covidence PRISMA Flow chart of search strategy) [30] with reference to steps for identifying and synthesising qualitative literature outlined by Shaw [31]. The process involved several stages: (a) a preliminary literature review (using library database) to support the development of a research question using the Context How Issues Population (CHIP) tool [32]; (b) a search strategy identifying key bibliographic databases and review of their thesaurus terms then developing a key word search; (c) registration of the systematic review on PROSPERO (ID: CRD42022339779); (d) running a search and exporting material to Endnote and Covidence to facilitate screening and develop a PRISMA flow chart [33]; and quality appraisal of extracted papers via an adapted 12 question Critical Appraisal Skills Program (CASP) tool [34] to denote studies as high, medium and low quality; (e) data management via Microsoft Excel; and (f) determining the most appropriate method of synthesis based on the review question.

### Inclusion criteria

For broad inclusion, a variety of English language sources, focussing on AN or atypical AN with minimum 3 years illness duration were considered i.e., qualitative, mixed methods or case reports, journal articles, books, theses, grey literature and expert libraries. Atypical AN is a recognised form of AN [35] and given there is no upper limit of BMI in the Diagnostic and Statistical Manual (DSM-5) [36], clinician judgement is applied. Inclusion criteria encompassed: adult population where more than 90% of participants had an illness duration of over 3 years, in line with proposed SE-AN criteria. Authors were contacted for further information if illness duration was not available within the paper and given a month to respond.

### Search strategy

The search strategy was designed to optimize the number of papers, anticipating a paucity of research into those living with SE-AN. The search encompassed all years from five bibliographical databases: psychINFO & CINAHL (Ebscohost), SCOPUS, MEDLINE & EMBASE (Ovid), a supplementary search (hand-search, citation tracking), consultation with subject experts and Google Scholar. Index terms, subject headings and keywords (see Additional file 1) were developed in consultation with the university librarian (see acknowledgement).

### Methodology for evidence synthesis

Meta-ethnography was used to synthesize findings across the extracted studies to produce something ‘greater than the sum of its parts’ [37]. This analytic framework has been used for other meta-syntheses of eating disorders research [21, 24, 28]. Once familiar with the individual studies through engagement in repeated reading and the process of extraction of key quantitative data, the synthesis proceeded via an iterative process that involved a series of repetitive and overlapping phases. These included: (a) separating the three foci (phenomenology, treatment, and recovery) between and from within papers (b) extracting the theme name or “headline” from each of the studies; (c) illustrating this with a detailed “descriptor” using the original authors own words in context; and (d) providing at least one verbatim “exemplar” of the theme from the perspective of the experiencing person (See Table 1).

**Table 1:** Example—Interpretation of themes—*Heading, Descriptor, Exemplar* process

**Author Date**	**Heading**	**Descriptor (authors words)**	**Exemplar (participant quote)**	**3**^**rd**^ order construct(s)*	**pg**
**Hannon et al. 2017**	**Treatment experience**	Novel intensive community treatment was preferred to the inpatient care – more reflective of real world, therapeutic alliance being held and the slow pace no pressure was key	everyone in the team was so compassionate, nobody judged me…I think until you’ve built up trust with anyone its hard to make the changes… if I hadn't had X who knew me… helping me carry on, then I couldn’t have got so far	**Treatment help versus harm** **Hope**	**287**
	**Function of AN**	a positive presence in an unhappy life… helpful… feel better.. pride… comfort / safety..distraction from uncomfortable feelings More acceptable… less isolated	*Really low… lose a bit of weight feel better about myself for just a second*	**Theme 2a meaning of AN to Self**	288
	**Self criticism v’s self acceptance**	self blame and guilt for having AN versus caring, accepting and compassionate towards self	*..my own fault… wasn't good enough… im the problem* *At first that was so alien to me* (self-kindness*)… never thought about doing anything nice for myself…do for other people… but not for me*	**Theme 4b**	**288**
	**Isolation versus connection**	isolated, empty and lonely because unable to trust others	*I remember feeling quite lonely…felt I didn't want to be around anybody else… like I had caused all the problems… im scared of being alone all my life*	**Theme 3**	**288**
	**Hopelessness versus Hope**	long time in treatment with little change reduced hope more hopeful if specific obtainable goals	*I feel like really, really hopeless and its like soul destroying. Because I can't see anything changing*	**Theme 2b** **Hope**	**290**
	**Stuckness versus Change**	stuck, trapped, afraid and conflicted, frustrating and tormenting place. A tipping point	*…its really, really frustrating to understand… at the stage I don’t want to change. I want to change…. No I don’t want to change… I want to get better..but don’t want anything to change… its just infuriating*	**Theme 2b**	**290–1**

The *headline, descriptor, exemplar* (HDE) method defined for this review [38] enabled integrity in the progressive interpretation, facilitating communication between synthesizers and subsequent thematic comparisons. For the synthesis of translations in the final stage, themes were then compared, contrasted, and rolled into one another, such that overlapping meanings could be denoted to one “meta” theme, for communication of findings.

A meta-theme was the final outcome of the translation between studies and evolved in up to 8 iterations. The first author (LK) was responsible for the generation of preliminary meta-themes, which were discussed with both secondary authors, and by way of encompassing multiple perspectives to ameliorate the risk of bias. All three authors collaborated to develop the thematic map. Once reviewing the individual foci, it was then possible to translate the subjects into a meaningful whole, whereby the AN, its treatment and recovery could be viewed as a *process* in a conceptual map.

### Reflexivity

The phenomenological lens impacts the process of synthesis, informed by collective personal and professional life experiences of the researchers. Of relevance for this review, researcher LK has worked clinically with people experiencing eating disorders, encompassing end stage care, as an accredited practising dietitian and, professionally registered psychotherapist / counsellor, specialising in Gestalt Therapy interventions, within an existential, relational, paradigm. JC is a Clinical Psychologist, Dietitian and academic in Clinical Psychology. Her research and clinical work seek to prioritize the voice of the experiencing person to inform the development of a broader range of ED treatment interventions that have scope to be flexibly tailored to the needs and preferences of the experiencing person and their family. PH is a clinical academic Psychiatrist formally trained first in psychodynamic psychotherapy and then in cognitive behaviour therapy. She has experience of caring for many people with SE-AN in general hospitals and outpatient private practice setting, was a lead investigator on a SE-AN clinical trial [14], and lead author on Australian guidelines [8] endorsing the need for new person centered and flexible approaches in care.

## Results

### Procedure for extraction

The initial screening by title and abstract resulted in 242 papers. Full text reading of these papers (100% LK, 20% PH, 20% JC) identified 78 did not specify illness duration. Authors of these papers were contacted and 29 replied, with six then meeting inclusion criteria. Full text reading and author clarification resulted in a total of 36 papers [39–74] meeting criteria and were extracted for this meta-synthesis (see Fig. 1).

### Study characteristics

Of the 36 extracted papers, the primary subject focus included phenomenology (*n* = 25), treatment experiences (*n* = 13), and recovery (*n* = 4), with overlapping foci within papers positioned within each relevant category for analysis. The characteristics of the included studies are outlined in Table 2.

**Table 2:** Characteristics of the 36 studies included in the Metasynthesis

**Source Paper & Foci**	**Year**	**Country Setting**	**Sample: size (N),** **%Female(F) /male (m), mean age (M) and range (R) (years)**	**Illness duration (yrs), diagnosis (Dx)—self or method), author stipulation**	**Treatment Status (TS:Y/N sought treatment)** **Treatment Type (T), Description (D), number (N)**	**Recruitment**	**Data Analysis**	CASP RATING *** = HIGH QUALITY
Arkell & Robinson (P)	2008	UK	*N* = 11 *F* = 91% age = 37.7yrs	> 10yrs, Dx ICD-10, SEED-AN	TS = Y (all) T,D,N unknown	Community treatment setting	‘coding’ (Serpell 1999)	*
Antoine et al. (P)	2018	France	*N* = 5, *F* = 100% age = 35ys (*R* = 29-46yrs)	M = 14.4y (*R* = 10-18yrs), Dx = U, chronic AN	Unknown	Clinical	IPA	***
Broomfield et al. (P)	2021	Australia	*N* = 11, *F* = 100%, age M = 41.6 (*R* = 29-66yrs)	M = 26.2yrs (*R* = 7-53yrs) Longstanding AN	TS = 10/11	Community	Narrative TA	***
Blackburn & O’Çonnor (P & TE)	2020	Ireland	*N* = 6, *F* = 100%, age M = 39.4yrs (22-44yrs)	*R* = 8-28yrs, formal Dx, Longstanding AN	TS = All > 2 treatments, all = OP, 5/6 = IP	Community	Not specified	***
Chinello et al. (P)	2019	Italy	*N* = 5, *F* = 100%, age M = 46yrs (40-54yrs)	Dx = DSM-5, M = 29.2yrs (*R* = 23-37yrs)	n/a	Community	IPA	**
Cardi et al. (P)	2018	UK	*N* = 90, *F* = 97.8%	Dx = DSM-5, M = 8.4y, *R* = 0-46yrs	TS Y (all), 37% IP, 100% OP	Community	TA	*
Dalton et al. (TE)	2022	UK	*N* = 15,	M = 15.05yrs, Dx = DSM-V, SE-AN	TS Y (all > 2prior treatments, M = 2.47, IP *N* = 2.31stays), rTMS	Clinical	Inductive—Elo and Kyngas (2008)	*
Dawson, Rhodes & Adams (R)	2014	Australia	*N* = 8, *F* = 100%, *R* = 31-64yrs	M = 15.5yrs, *R* = 9-44yrs, Dx = DSM4	TS 100% = Y,	Community	Narrative TA	***
Eivors et al. (TE)	2003	UK	*N* = 8, *F* = 100%, M = 25.75yrs, *R* = 21–43 yrs	M = 6.75, *R* = 3-20yrs	TS 100% = Y, IP = 50%, OP = 100%	Clinical	Social Constructivist GT	**
Espeset et al. (P)	2012	Norway	*N* = 14, M = 29.1yrs, *R* = 19-39yrs	M = 10yrs, Range 3-25yrs Dx = 100% DSM-4,	TS 100% = Y, IP = 57%, OP = 100%	Clinical	GT (Corbin and Stauss)	**
Fox & Diab (P) (TE)	2015	UK	*N* = 6, *F* = 100% Age M = 29.5yrs *R* = 19 to 50yrs	M = 7yrs, *R* = 6-23yrs. Dx = DSM-4, Chronic AN	TS = 100% > 2 psychological therapies	Clinical, Two eating disorder services	IPA	***
Foye et al. (P)	2019	UK	*N* = 5, *F* = 80%	*R* = 5-20yrs, Dx = 4/5 formally, SEED-AN	TS = 4/5, IP = 60%, OP = 80%	Clinical	TA	***
Hannon et al.(P) (TE)	2017	UK	*N* = 5 *R* = 23 -30 yrs, *F* = 100%	*R* = 4-11yrs, Dx 100% ICD 10, SE-AN	AN Intensive Treatment Team model: intensive community-based care (> 2years)	Clinical Treatment settings	IPA	***
Joyce et al. (P) (TE)	2019	UK	*N* = 8, *F* = 87.5%, M = 44yrs, *R* = 20-64yrs	*R* = 10-40yrs, SE-AN	TS 100% > 2, IP = 62.5%, OP = 100%	Clinical	NA	***
King-Murphy (P)	1997	Canada	*N* = 6, *F* = 100%,	M = 12.8yrs, *R* = 6-18y	TS = 100% > 2, IP = 67%, OP = 100%	Clinical	Van Mannen’s phenomenology	***
Kolnes (P)	2016	Norway	*N* = 6, *F* = 100%, M = 32.5y, *R* = 23-50yrs	M = 12.8yrs, *R* = 6-18yrs	TS = 100% Y,	Clinical residential	IPA	***
Kyriacou et al. (LE)	2009		*N* = 6 *F* = 100% M = 26.8yrs R-20-36yrs	M = 10.7yrs, *R* = 4-22yrs	TS 100% Y	Clinical	TA	**
Marchant & Payne (TE)	2002	UK	*N* = 5, *F* = 100%, *R* = 22-38yrs	*R* = 4-20yrs	TS = 100%, IP = 20%, OP = 100%, psychodynamic, Gestalt, CBT, PCT	Community	Heuristic (Moustakas 1990)	*
Marzole et al. (P)	2015	Italy	*N* = 34, *F* = 100%, M = 25,6yrs, *R* = 18-40yrs	M = 9yrs, *R* = 4-14yrs, Dx 100% = DSM	Psychodynamic, DP	Clinical DP	Coding (Serpell, 1999)	*
McCallum & Alaggia (P)(R)	2021	Canada	*N* = 19, *F* = 95%, M = 50.6yrs, *R* = 40-64yrs	M = 19.2yrs, *R* = 11-40yrs,	TS = 100% Y	Community	GT	***
Musolino et al. (P)	2020	Australia	*N* = 5, *F* = 100%, M = 25.8yrs, *R* = 27-52yrs	M = 18yrs, *R* = 10-30yrs, Dx formal (100%), SE-AN	TS = 100%Y	Community	GT	**
Lyons (P)	2018	UK	*N* = 7, *F* = 0%, M = 28yrs, *R* = 23-34Yrs	M = 11, *R* = 4-19yrs	TS = 100% Y, CBT, Schema, Psychodynamic, OT, Dietetics	Community	Narrative	***
Ostermann et al. (T)	2019	Germany	*N* = 1,(F), Age 38yrs,	Duration 30 years,	IP > 4, Yoga	Treatment sample	Not-specified	*
Patching & Lawler ®	2009	Australia	*N* = 20, *F* = 100%, *R* = 24-52yrs	*R* = 4-17yrs, Dx formal = 55%,	formal treatment IP or OP = 55%, self-help = 45%	Community	Narrative TA	*
Ramjan et al. (TE)	2017	Australia	*N* = 6, *F* = 100%,M = 26.8yrs, *R* = 18-38yrs	M = 5.4y, *R* = 1-10yrs (1 pt < 3yrs) Dx = Self report	Unknown	Community Treatment – Peer Support	TA	***
a. Rance st al. (R)	2017	UK	*N* = 12, *F* = 100%, M = 31.5yrs, *R* = 18-50yrs	M = 13.29yrs, *R* = 2-28yrs (91.6% > 4yrs)	CBT, Analytic, Psychodynamic, Integrative	Purposive community	TA	***
b. Rance et al. (TE)	2017	UK	As above	As Above	CBT, Analytic, Psychodynamic, Integrative	Purposive, Community	TA	***
Robinson et al. (P)	2015	UK	*N* = 7, *F* = 71.4%, M = 50yrs, *R* = 40-59yrs	M = 32.1yrs, *R* = 20-40yrs, Dx DSM5, SEED-AN	IP = 86%, OP > × 2,	Clinical	TA	***
Ross & Green (P)(TE)	2011	UK	*N* = 2 Age: 18 + years *F* = 100%	Duration > 5yrs, Chronic AN	Psychodynamic inpatient treatment	Clinical (ED service)	Narrative TA	***
Strand et al. (P) (TE)	2018	Sweden	*N* = 16, *F* = 94%, M = 31Yrs, *R* = 18-56yrs	M = 15yrs, *R* = 3-42yrs, Dx DSM-5,	TS = 100Y Unspecified	Clinical	Inductive (unspecified)	***
Stockford et al. (P) (TE)	2018	UK	*N* = 6, *F* = 100%, M = 36yrs, *R* = 33-48yrs	M = 20.6yrs, *R* = 14-28yrs, Dx ICD-10 (all), SE-AN	TS = 100%Y	Clinical	IPA	***
Thoresen et al. (TE)	2021	Norway	*N* = 1 (F), Age = 23yrs	Duration 7yrs, Dx = formal	Symptom-focused treatments (7yrs) Psychodynamic (12 sessions)	Treatment setting	IPA	*
Trondalen (TE)	2003	Norway	*N* = 1(F), Age = 26yrs,	Duration 6 yrs,	Prior treatments = Y, IP × 2, OP 2.5 yrs, Music Therapy	Treatment setting	Not Specified	**
Williams et al. (P)	2016	UK	*N* = 11, *F* = 100%, M = 28yrs, *R* = 18-60yrs	M = 13.5yrs, *R* = 5-48yrs, Dx = DSM5 (all),	Unknown	Community plus 2 ED clinics Theoretical sampling	GT	***
Wright & Hacking (TE)	2012	UK	*N* = 6, *F* = 100%, *R* = 21-44yrs	M = 11yrs, Dx formal	OP = 100%, treatment M = 11 years	Day care services,	Van Manen 1990	*

*Key*: *IP* Inpatient, *OT* Outpatient, *DP* Daypatient, *GT* Grounded Theory, *IPA* Interpretative Phenomenological Analysis, *TA* Thematic Analysis, *P* phenomenology, *TE* Treatment Experiences, *R* Recovery, *U* Unknown

This synthesis of 36 papers, includes the voices of 382 people with longstanding AN, (Atypical AN was undifferentiated) with 8.6% males (see Table 2). The average age of the sample was 33.2y (range of means 23.5 – 49.9y) with a mean illness duration of 14.56y (range of means 8 – 29y); whereby > 90% of participants had an illness duration of more than 3 years. While the sample cannot be empirically defined as SE-AN, it represents an illness duration far exceeding (5 times longer) the proposed minimum with the majority (*n* = 31 or 86%) reporting at least two treatment attempts, with data related to treatment unavailable for the remaining five studies. It is a group identifiable as “SE-AN – we know it when we see it” ( [75] p 1315.) or in this case, hear it, from participants, in their own words.

Given criteria for SE-AN remain un-tested, characteristic labelling of the sample by the authors of constituent papers was of interest (Table 2). It was found that one third of all papers (*n* = 14) adopted labelling indicative of an enduring eating disorder with most papers (*n* = 9) using the term “severe and enduring anorexia nervosa” [39, 45, 50–52, 64, 66, 67, 73] and the remaining papers referring to “chronic” [40, 49, 65] or “longstanding” [41, 42].

A summary of all themes in the papers is included in Additional file 2 Box 1.

### Quality assessment of selected studies

Quality appraisal of all the extracted studies utilised an adapted, 12 item CASP tool [34]. A score of high, medium or low risk of bias was assigned by the first author (LK) for 100% of papers, and 10% (assigned randomly) each by PH and JC. Discrepancy in category allocation / scores were resolved by discussion. More than half (*n* = 20) of the papers were allocated a ‘high’ quality score, with 25% allocated as low quality (see Table 2). Consistent with the aim of inclusivity, papers were not excluded based on quality. However, methodological rigour / transparency impacted quality scores, such that fewer verbatim quotes were available to cite in this meta-synthesis. Therefore, by virtue of the translation process of synthesis, their use was limited, relative to papers of higher rigour.

### Synthesis of results

The individual concepts of interest – phenomenology, treatment, and recovery, each generated several preliminary meta themes. This meta synthesis generated four meta themes with a series of sub-themes and three themes that cut across these meta-themes. Meta- and cross-cutting themes related to the phenomenology of SE-AN, as lived and over time, encompassed treatment experiences as a part of participants’ life worlds, and recovery. The themes and sub-themes, their presence across the extracted papers and the four cross cutting themes are summarised in Table 3: Themes, sub-themes and cross cutting themes. It became apparent for the translation that the higher order themes and sub-themes constitute recursive, dynamic, temporal ‘phases’ and are discussed in these terms.

**Table 3:** Themes, sub-themes and cross-cutting themes

**CROSS CUTTING THEME**	**THEME #**	**META THEMES TITLE**	**(N)**	**CROSS CUTTING THEMES**	
**SHIFTS IN CONTROL**	**1**	**VULNERABLE SENSE OF SELF:** ‘*Hesitance to Exist*’	28	**HOPE vs HOPELESSNESS**	**TREATMENT – HARM Vs HELP**
	**2**	**INTRA-PSYCHIC PROCESSES**			
	2a	Meaning of AN to Self: ‘*Double Masquerade*’	19		
	2b	The Disappearing Self	25		
	**3**	**GLOBAL IMPOVERISHMENT**	24		
	**4**	**INTER-PSYCHIC, TEMPORAL PROCESSES: CHANGE AND RECOVERY**			
	4a	Opening the Self to Other(s)	19		
	4b	Re-integrating Self	18		

Given the enduring nature of SE-AN, it was evident from the frequency of themes that the most cited aspects of the experience related to intra-psychic or internal processes. This reflects the higher number of papers related to phenomenology *n* = 25, which extended to the phenomena of being in a relationship [40] or an expectant mother [43]. There were 13 papers with a treatment experience focus, half of which [45, 51, 68, 69, 72, 74] reported on novel, non-evidence-based interventions. Relatively fewer papers cited participant experiences of temporal processes of change and recovery, whereby only four of the 36 (8.6%), had the specific focus of recovery [46, 58, 61, 63].

## Themes

### Meta-theme 1: interpersonal phenomenology

#### 1a Vulnerable sense of self: *hesitance to exist*

The vulnerable sense of self, from across 28 studies [39–46, 48–50, 52, 53, 55–62, 64–68, 70, 73, 74] is a composite of suffusive shame and intrapsychic factors ( [62] p 130). Here, shame processes fracture a person’s relationship with themselves encompassing feelings, emotions, body, identity and alienating them from the self and others. This is exacerbated by the AN experience itself through ongoing encounters of being misunderstood by others, including stigma related to AN.

##### Extracts 1

*All my life I was made to feel like a nobody. I was moulded by my parent’s little world[...] with my first husband it was the same (King-Murphy et al. p 90 *[53]*)*

*…as long as people know I have anorexia I see a difference in the way they look at me[...] Judgement, prejudice, distance right away[...] treat me as a person with brain damage (Schut et al p 9 *[66]*)*

*I felt like his (husband’s) puppy dog on a leash and every time he would tug me back when I* *would be out too far[...] I realized I was slowly killing myself to let him live[...] He took my freedom[...] Then he almost took my life (King-Murphy p 82 *[53]*)*

These extracts highlight some of the ways that participants experience a vulnerable and fractured relationship with self and others. In these extracts, participants’ sense of abandonment and perceived invisibility to significant adults around them are evident, where they felt controlled, thereby contributing to an impoverished sense of self. The relational pattern continued as a legacy in future relationships, and due to their illness through broad experiences of stigma.

Shame, found in participant accounts across 19 studies [39–44, 49, 52, 53, 58, 61, 62, 64–68, 73, 74], was manifest in a pervasive sense of unworthiness. For example:

##### Extracts 2

*Suppose mainly it's just my own self-worth my own self-esteem and just like my, the way that I view myself and the value that I've got in my life I suppose is just like really low, so it's hard to look after myself properly, I guess. When you don't think that you deserve anything or, yes, it's really hard to just take care of yourself and say you are worth looking after and feeding yourself (Stockford 2017, p 132 *[67]*)*

*I’m not allowed to be here. That I’m[…] I shouldn’t really have been like, born. In a way I’m, I’m just a burden to the world (Blackburn et al. p 435 *[42]*)*

A fear of vulnerability, “exposed […] seen as vulnerable” (Kyriacou et al. 2009 p 849) grounded in mistrust and consideration of themselves as fundamentally flawed, defective, and unworthy is seen in these extracts. Shame about who they were as a person extended for some to a sense of being unworthy of treatment, “guilty taking up the nurses time” (Robinson et al. p 323 [64]) and their foundational needs, emblematic in their eating (“it’s hard to look after myself”). AN was viewed as the means to morally repair the self and to ameliorate internalised shame;

##### Extracts 3

*Anything that passed the due date in the fridge I wouldn’t give it to anybody else, but I would have it for me. I used to have this attitude like rubbish for rubbish (Robinson et al. pg 320 *[64]*)*

*I need to go through a lot of hurt [...] to be a better person* (Blackburn et al p 435 [42])

These participant accounts exemplified the SE-AN experience as a performance of this worthlessness (“rubbish for rubbish”). The studies revealed accounts of a vulnerable sense of self through a fractured relationship with the self, a perceived low self-efficacy with regards to feelings and emotions and permeating sense of un-worthiness. In response to this embodied unworthiness, the solution for some was to detach from their bodies;

##### Extracts 4

*I was invisible to the world; I was a ghost, voiceless. No matter how bad it got I couldn’t get it out or show it [...] Whether it was visible ribs or scars on my skin, I just didn’t know how else to express it and this was my way of showing that I was hurting (Foye et al 2019 p 332 *[50]*)*

*Live in this kind of ethereal world[…]pure[…]holy[…]detached from bodily concerns and needs. Rance et al. 2017 p 132 *[62]

Identified in participants’ accounts was a need to disconnect from the embodied distress, as the home of their feelings and emotions, which were experienced as intolerable and threatening. Instead, physical pain was desired over emotional pain and the body was used as a language of expression of hurt and pain.

Finally, being in relationships and engaging in the world (including in treatment) was undermined by the shame, un-worthiness and fear (of abandonment) where participants expressed a hesitancy to exist;

##### Extracts 5

*We hold ourselves back from that [creating relationships]; won’t allow ourselves (Foye et al. p 335 *[40]*)*

*Think it's kind of a BMI thing is quite deceptive in terms of its use by medical professions in terms of determining who needs help and therefore it contributes to the anorexic thinking that ‘oh well I'm not actually ill enough I don't actually deserve the help, I need to go out and be more anorexic to get more deserving and achieve more whatever (Stockford et al. p 135 *[67]*).*

Within the *vulnerable sense of self*, timing of treatment was critical for some participants (“wasn’t the right time for me”), where people were not ready to ‘*open the self to other*’.

##### Extract 6

*I had dozens of hospital admissions with no success [...] treatment [...] like banging my head against a brick wall [....] Scare tactics [....] if the clinicians told me what to do id think well screw you I’m not doing that [...] us and them staff was telling me there was no hope for me [...] thought I was stupid and ignorant. Dawson et al p 499 *[46]

To this end, treatment needed to respect a person’s autonomy “freedom to make decisions taken away” *(*Lyons 2019 p 106 [59]*)* in order to minimize resistance (“screw you”) and further isolation (“thought I was stupid and ignorant”), building hopelessness (“like banging my head against a brick wall”).

Of the most disturbing findings in the voices of this study, is that where a person turned to find helpful healing, they encountered treatment relationships that mirrored problematic formative relationships. With potential to reinforce psychopathology of isolation, shame and vulnerable sense of self.

##### Extracts 7

*Male doctors very abusive and in a sexual way and that doesn't help they let me down. (Joyce et al 2019 p 2078 * [52]*)*

*So it was refreshing to have people that actually did present as caring and I think for an illness like this where people have really low self-esteem anyway and feel, most of them feel not worthy of receiving treatment and that they're being a burden on other people and they feel they've brought it all on themselves and actually it's probably not helpful to have that reinforced [...] more important than the food to be honest to have people that care for you and accept you and that don't look down their nose at you and ‘ya know see you as a worthy human being that's an individual and that can spend time with you  not just go shovel you full of cheese and fattening foods with no humanity (Stockford et al. 2018 p135 * [67]*)*

These accounts of a vulnerable sense of self and treatment not meeting a person’s needs, appeared to provide a foundation for the perceived curative allure of AN and progression the next phase of illness.

### Meta-theme 2 – intra-psychic processes

#### Theme 2a: meaning of an to self: double masquerade

Across 19 studies [39–41, 44, 47, 48, 50, 51, 54, 55, 57, 59, 60, 62, 64, 66, 67, 70, 73], participants talked about how AN had become meaningful for them, particularly where it was experienced as a solution to addressing a vulnerable and fractured sense of self (Meta-theme 1). For example:

##### Extracts 8

*I use it [AN] to keep life ticking over and keep the happy smiley face on [...] Competent image [...] I didn't use that then I think I’d be in the point where I can’t get out of bed in the morning depression wins so it’s the lesser of two evils really (Rance et al 2017 p 132)*

*it’s like a protective thing and it feels like round my heart [...] it made you feel more erm, secluded from the world really, in this like, in this total bubble of your own making [...] if you were to take them all down, I don’t know, I think I’d feel really, really vulnerable […] you feel safe if you know what to expect if you stay on this sort of a routine (Musolino et al p 6 *[73]*)*

*Anorexia offered this really clean, pure, serene, space that really contrasted to all that messy, ugly, nasty, out of control stuff…it’s a safer place to be (Broomfield et al. p 6 *[41]*)*

These extracts exemplify how AN functioned adaptively in the context of a ‘vulnerable self’ where a person is extended beyond the level of coping or self-support (meta-theme 1). The functionality, described as a “wonderful thing that actually works” *(*Foye et al. p 330 [40]), varied for people. It offered intra-psychic relief from “vulnerability” (Extract 8) and interpersonal distancing by projecting an exterior of “happy, smiley” emotional “competence” (Extract 8). It represented empowerment through lack of needs, allowing a person to punitively “push myself to the extremes[…]without needing anything” (Robinson et al. p 320 [64]). AN brought a sense of control via a “serene space” and “safe place” within a perceived “messy” world. It was a source of pride to mask internalised shame “Keeps my demons locked away, of inadequacy, failure, weakness…” (Arkell & Robinson p 653 [39]) and restriction served as a way avoid, suppress, express and communicate emotion; “There’s a mash of emotions squashed in together…I found myself looking for a way to dull […] numb […] quash all of those emotions—to avoid feeling, to get to a place of not feeling… this was my way of showing that I was hurting” (Foye et al. pp. 331–332 [50]). For others, the AN was compatible with their view of themselves and self-hatred “When I hate myself it’s a way to treat myself badly. It is like a self-destruct button, and this has been what I wanted sometimes” Arkell and Robinson 2008 p 654 [39]*.* In this sense, AN is considered ego-syntonic with a person’s uncompassionate view of themselves and compatible with a vulnerable sense of self.

We have termed this process *functional adaptation* from a synthesis of ecological psychology [76] and existential perspective [77] that is designed for ‘survival’ of the ‘human organism’ [78]. This *functional adaptation* is enlisted in AN as a “beneficial transformation”. It assists the person to respond effectively to life [79, 80] to achieve homeostasis or harmony in their being. Representing an effort to preserve oneself, and to survive. The meaning or functionality varied widely for individuals for example, providing a sense of order and control, empowerment, inter and intrapersonal distancing, avoidance, pride, punishment, protection (against vulnerability or engagement in life), management of painful emotions and safety in predictability and refuge.

The functional aspect of AN, understood as a *creative,* functional adaptation to help the person to survive, was positioned to ‘lose its shine’ for people over time. So ensued a complex, ambivalent relationship with AN, where it offered ‘refuge’ but was also experienced as ultimately destructive. As exemplified through metaphor, for example a “spider”, “sniper”, “monster”, “devil”, “evil twin”, and “abusive partner”.

##### Extracts 9

*It gives you like a split personality. Good and bad, positive yeah, it’s like, it’s like your evil twin type of thing[…] I guess the only way I can describe it is as an abusive relationship, you often think why do you stay in that relationship but because you almost feel helpless without it. Yeah, it’s like an abusive partner, you, you know, you don’t like it but it’s, they promise to look after you so you believe them (Williams, King and Fox p 221 *[70]*)*

*builds a web so no one can come close to the real him, because alone he stands[...] wary[...] yes, its not enough with just the spider[.…] needs to have something around himself (Thoresen et al 2021 p 187 *[68]* )*

*[...]. It is a sniper who approached me slowly and before I knew it, it was ingrained in my life (Schut et al. p 7 *[66]*)*

Across these studies participants used a range of metaphors for AN. This captured a complex social reality that included the polarity of protection and harm as they sought to navigate the world with a pseudo-wholeness and self-organisation. In honour of the multiple metaphors offered by participants, a metaphor offered for this theme is the ‘double masquerade’. For the person, the mask is the veneer to hide the apparent darkness and shadows within the self to “keep the demons locked away” (Arkell & Robinson p 653 [39]) and “smile” through the “pain”, concealing the perceived defective, “needy” self, within. For AN, the masquerade is the ‘opposing truth’, described as “split personality”, “evil twin”, the “good and bad” – the “monster behind the mask” (King-Murphy p 82 [53].

There appeared also to be a dual reality associated with treatment whereby it was seen as mirroring the punitive aspects of AN “being trapped not only in the hospital environment but also in the illness itself, it was desperation” (Ross and Green 2011 p 114) versus support and allegiance (Extracts 10). Also, dichotomous with regard to intensity, with a perceived abandonment in the lack of continuity in care post discharge going from being “very, very intense to very hands off” (Stockford et al. p135 [67]), and abandoning.

##### Extracts 10

*there probably is an atavistic sense of self-punishment and lack of worth associated with ED, that the structure and nature of inpatient treatment exacerbates. (Joyce et al., 2019. p 2075 *[52]*)*

*When you are in hospital you can battle it because it’s not just one-on-one you’ve got you and a whole team against anorexia, you are all fighting it but as soon as you leave hospital, and you get home it’s just one-on-one again and you are bound to lose kind of thing[…] it’s one extreme to another you need that integration probably less time inpatient more time ya know half way there[…]half way house kind of thing. Stockford et al. 2018. p 135 *[67]

*I felt like they were ganging up on me. Bringing me places I did not want to go. Forcing me to eat, forcing me. That was really, really bad. They could not understand. (King-Murphy 1997, p 86 *[53]*)*

#### Theme 2b: the disappearing self

As AN dominated, a phenomenon discussed in 25 papers [39–42, 45–47, 49, 51–53, 56–67, 70, 73, 74], it became a solution that temporarily held the person’s identity intact. However, in doing so, it also pervaded their sense of themselves, leading to harm, dislocation from others and disconnection from oneself and their life – *The disappearing self.*

##### Extracts 11

*Like you’re slipping into a whole different world[…].like you’re stepping out of your body, you’re looking at yourself and you’re doing these things and like even though I felt like I had control over it there were times when I really didn’t think I had any control of it at all (Rance et al. p129 *[62]*)*

*insignificant and without value. And that I don’t, that it makes me feel invisible maybe (Thoresen et al. 2021 p 188 *[68]*)*

These SE-AN experiences speak to a totalisation of the self by AN. The extracts exemplify how AN pervaded the self, functioning to separate the self “slipping into a whole different world” such that the “real world becomes more and more alien and difficult to remain in” (Arkell & Robinson 2008 p 654). As AN over-shadowed the self, the self, disappeared “I was more like a shell” (Williams King and Fox 2016 p 219 [70]) and AN was experienced as “part of me”. The shift from being offered an organising sense of control to being controlled appears to happen in this phase.

##### Extracts 12

*Living with AN is like having a monster inside of you. It consumes you[…]Can’t escape[…] Complete control of your whole life[…] This monster takes over[…] It has all the control; you have none. (King-Murphy p 82 *[53]*).*

Participants used metaphor to illustrate the sense that they had been taken over by a “monster inside” (AN) and “consumed”; unable to “escape”, feeling even more “out of control” than they did when their initial survival felt under threat, predisposing the AN, leaving them “invisible”.

Some people gradually built insight about the shifts occurring in relationship with AN—from a source of perceived control (functional adaptation) to being totally controlled by AN *(disappearing self*). For others the realisation of loss of control became a source of despair in which death “that’s when I wanted to die” felt the only escape, or prompted engagement in treatment when they recognised that they were no longer in control of their illness;

##### Extracts 13

*[.…]that’s when I wanted to die […] I was a slave, I wasn’t in charge any more, didn’t dare stand up was afraid of everything. I was so tired[...] I had to eat vomit eat vomit that’s how my days passed (Schut et al 2022 p7 *[66]*)*

*was devastated, I couldn’t go on like this [living with an eating disorder]. It was scary but I gave myself one more chance at recovery. If that didn’t work I couldn’t continue living like this [suicide]. Patching & Lawler 2009, p 16 *[61]

Treatment was perceived as surrendering of control and was a mixed experience for people and the absence of trust undermined engagement in treatment and progress;

##### Extract 14

*like a prison[…] loss of liberty[…]. Freedom to make decisions taken away [….] Being on a unit was the worst experience of my life ever (Lyons 2019 p 106 *[59]*)*

Limiting treatment to one aspect of a person (cognitive) was experienced as rigid and minimizing of the vulnerable person within. Worse still, where a preconceived idea of the illness was imposed onto a person, at the expense of their unique experience, this fuelled their symptoms;

##### Extracts 15

*It felt quite rigid and it was like ‘If you understand you have your thoughts and your feelings are reflecting [them] and challenging your negative thoughts then you will get better, and if you’re not getting better you’re just not trying hard enough[…]diagnostic tick box a thing with problems [.…] an object. Rance,Moller & Clarke p 588–89 *[63]

*he was like oh you can't be that bad because you’re not throwing up in bags, you’re not hiding it […] when I started seeing him I was bingeing and being sick about twice a week […] by the time I finished with him I was throwing up all day everyday. Rance, Moller& Clarke p 589 *[63]

As well as consideration of maintaining some control in treatment, timing was pivotal for people. If they did not feel ready or sufficient control through ownership of their treatment, there was a risk of causing long-lasting damage to the treatment relationship, including loss of engagement:

##### Extracts 16

*she showed me one of the patient bedrooms and she was talking about the spy hole in the bedroom […] and supervision after meals […] and I remember saying something about ‘Oh well […] I don’t make myself sick I just restrict’ and she said well “by the time we are finished feeding you, you might start!” and I drove off like a scene out of the dukes of hazard—there was like dust coming out of from behind (laughs. pauses). Needless to say I didn’t get admitted. I was petrified. Joyce et al 2019 p 2078 *[52]

*I’ll always be a little scared, since my very first experience was that I wasn’t actually allowed to discharge when I wanted to. So I’ll always have that fear, unfortunately (Strand et al. 2017. p 403 *[74]

In contrast, the importance of treatment choices, autonomy, readiness and titrated levels of control was highlighted in a self-admission program where this provided hope and reparation for people;

##### Extracts 17

*To get just a few days at the ward—shutting the rest of the world out, handing over choices and letting go of control. Strand et al. 2017. p 40 *[74]

*After I had made the conscious decision to get better, I started to do what the staff told me to do. It was absolute hell. I kept telling myself, though, that the devil was a sickness that was trying to take a hold of me, and I wasn’t going to let it, otherwise I would die Dawson et al. 2014 p 501 *[46]

Encouragingly, once people gained insight and awareness of the power struggle with AN, they could risk engaging in treatment on their terms. For some people, forfeiting AN, translated to some control in life whereby “…the illness had controlled my life and once I decided to [get better] I took back control of my eating[…] And then other things in my life felt more in control again” (Patching & Lawler 2009 p 16 [61]).

Collectively, the space given to AN in a person’s life was at the expense of others. It offered a functional solution, compatible with redemptive punishment and is ego-syntonic with perceived unworthiness, which at times was also reinforced by treatment. It is unsurprising the difficulty a “disappearing” person may experience in relinquishing their lives.

#### Meta-theme 3: global impoverishment

Across 24 studies [39–41, 44, 46, 48–51, 53–55, 57–61, 63–67, 70, 73] themes emerged where AN shifted from being an adaptive response to the environment to become a habitual, acontextual way of being in the world. For example remarking that the AN becomes “easy”, “natural” and “a script that you just can’t shake free of” Musolino et al. pp 5–6 [61]. When AN progressed to be a person’s predominant way of coping and left unexamined, it can become an automatic reflex, causing a *global impoverishment,* extending across all life domains—relational, physical, emotional, social, fiscal. Progressively, the person was faced with profound losses to the AN and the illness took on its own life, rendering it increasingly difficult to live alongside.

The immense impact of global impoverishment extended to loss of intimacy and relationships. Defending the anorexia “weird excuses […] lying […] problematic to let anyone get close” (Kolnes et al. p 8 [54]), led to guilt, related to dishonesty, and withdrawal; “I’ve got no one to be with, nothing special to do and it’s just horrible” (Robinson et al. p 321 [64]). This perpetuated isolation. Personal losses and absent milestones were highlighted when baring witness to siblings “move away” and on with their lives noting that “…I hadn’t ever been in a proper relationship” (Lyons p 255 [59]). For others who had partnered and had children the loss was of physical intimacy “nothing, no contact between us” (Antione et al. p 1847 [40] or losing the ability to care for the children they do have (King-Murphy p 83 [53]). Ultimately, the withdrawal extended to their own self, with disconnection from their thoughts and body (Foye et al. p 334 [50]), leading some people to feel estranged and “alien” (Espeset et al. p 458 [48]) to themselves.

Subsequent to unrelenting physical deterioration, engagement in pursuits outside of AN, such as work were expressed as increasingly difficult;

##### Extract 18

*I could not even talk clearly… I was not allowed to answer the phones any longer because the customers could not understand what I was saying. ( King-Murphy p 75 *[53]*)*

Being too physically unwell to engage in life outside of AN, further exacerbated isolation, eroding self-esteem limiting a person’s capacity to engage in treatment. The participants, already diminished to a slither of themselves, faced intense grief. The loss in overall quality of life may cause despair, to the point of attempting suicide;

##### Extracts 19

*[…] lost my job […] my flat […] just gone bankrupt […] I’m living with my parents now […] Anorexia a better substitute […] I have lost everything through the eating disorder absolutely everything…. (Stockford et al. p 133 *[67]*)*

*[predisposing unsuccessful suicide attempt]: I had no quality of life left (Musolino et al. p 6 *[73]*)*

**Fig. 2:**
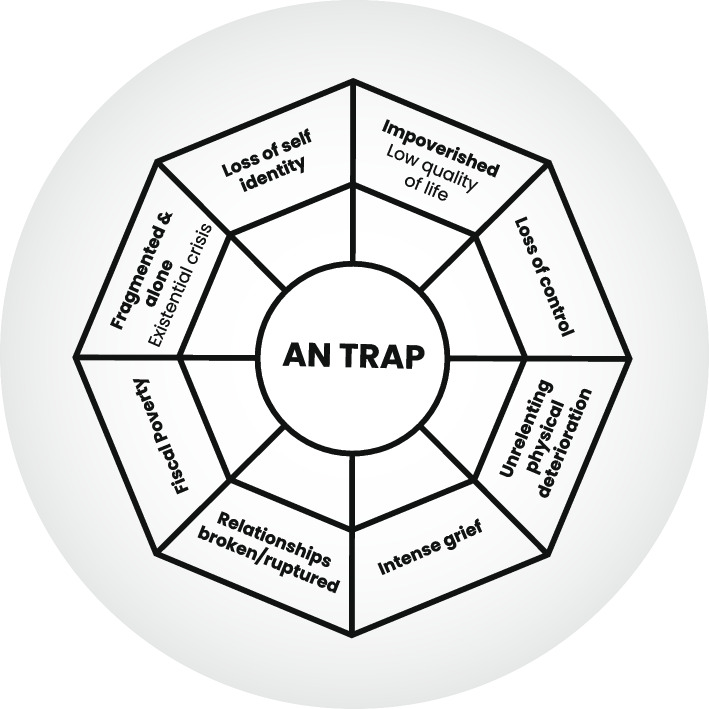
Conceptualising The AN Trap

**Fig. 3:**
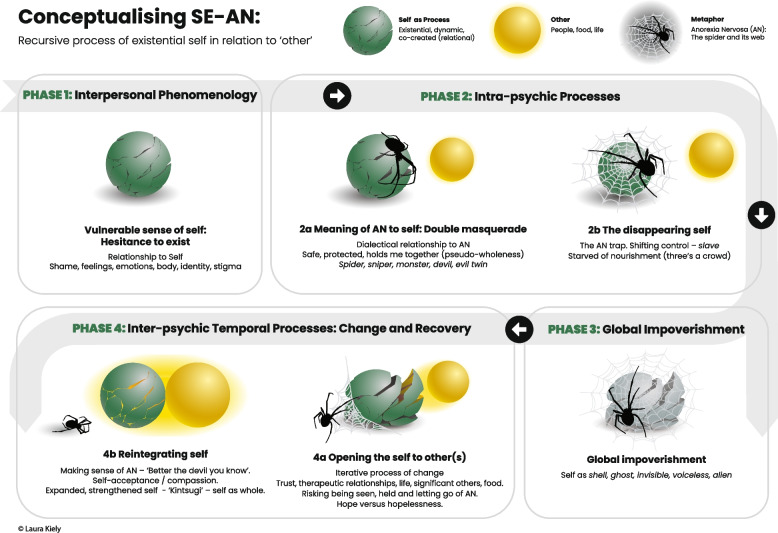
Conceptualising SE-AN: Recursive process of existential self in relation to other

This enmeshed relationship with AN, has been conceptualized using a participant’s metaphor of a spider’s web (Thoresen et al. 2021 [68]) and termed ‘The AN Trap’. This is an extension of Hay et al. 2016’s [81] original concept, expanded based on the voices in this review (Fig. 2) and placed in context later, in Fig. 3.

This state of *global impoverishment,* including loss of self to AN can lead to further entrenchment;

##### Extract 20

*I think just that anorexia gives you a sense of being that you wouldn’t have otherwise […] and I think without that you wouldn’t be, well, I wouldn’t feel like a person at all. (Williams et al p 221 *[70]*)*

The self becomes elusive “shell, ghost, invisible, voiceless, alien” and the world closes in, in parallel to AN taking on a life of its own. People may be unable to work, increasingly dependent on caregivers or financial welfare benefits, while intense loss and grief overshadow the person, who questions their very existence. A participant states;

##### Extract 21

*[...]in that way kind of the anorexia thing is self-perpetuating because [… ] I can't work because of Anorexia and low mood and so I don't have any kind of defining things when people ask you what you do, well not a lot really. I don't really do a lot and so it's almost like that allows the illness to become stronger because it feels like it's therefore quite an important role because you don't have another role and then it has it has to kind of compensate for all the things that aren't in your life like ya know a family or children, an academic career or any of those things ya know if you can't be really really any good at those things then being really really good at Anorexia would be a better substitute (Stockford et al. p133 *[67]*)*

It is in this phase of *global impoverishment,* the functional impairments which occur secondary to the functional adaptation in AN, become illness maintaining factors.

### Meta-theme 4: inter-psychic, temporal processes: change and recovery

It was notable that very few of the papers (*n* = 4) had the key foci of recovery [46, 58, 61],or a recovery sample [63]. Within treatment and phenomenology papers, we searched for processes of change, identifying a further 15 papers [42–44, 49, 51–53, 56, 63–69, 71, 72, 74] that contributed phenomenological insights. We found that *opening the self to other(s)* and *reconnection to the self* were key processes emerging as important to participants considering engaging in or reflecting on change and recovery.

#### Sub theme 4a. Opening the self and to other (s)

Opening the self to other – food, life, people, therapeutic relationships is a critical point in the recovery from AN, which is conceptualised in this recursive as a relational illness – where the disconnected self, risks isolation and alienation—‘it is hard to connect with others when you can’t connect with yourself’ (Foye et al. p 335 [50]). Self-sufficiency (“deal with the world on my own*”)* was underpinned by disgust in perceived neediness (“urg […] so needy”) (Blackburn et al. p 434 [42]). Honouring the inherent interwovenness of human existence, as built through trust and safety is fundamental to the process of inter-dependent expansion of self;

##### Extracts 22

*compassionate, not judged [….] until you’ve built up trust with anyone it’s hard to make the changes […] knew me […] helping me carry on( Hannon et al p 287 *[51]*)*

*need time to build trust in your therapist [….] I found that relatively easy to do, it was probably in six months or a year[...]I was able to feel that (Rance, Moller & Clarke p 588 *[63]*)*

If a person was received within the safety of trusting relationships from therapist, peer mentor, support groups or trusted other, hope builds and strengthens their potential for change;

##### Extracts 23

*I eventually found two amazing therapists who I stayed with until the end of treatment. One of them had had an eating disorder herself, and she was by far the most important therapist I had ever seen. It felt like she was reading my mind. She understood it and she instilled hope that I could get over it (Dawson et al p 501 *[46]*)*

*she’s like a little support angel on your shoulder […] she just makes you feel safe somehow. She makes you feel like she can hold you and the disorder, and no matter what happens she’s got hold of you and don’t worry ‘cos there is somebody there. (Wright & Hacking 201 p 111 *[71]*)*

Participants articulated the sense of feeling “seen” beyond their anorexia (“sees me as a whole person”) (Rance, et al. p 586 [63]) and held (“hold you and the disorder”), which for some involved commonality of a shared history (“had an eating disorder herself”) and met “genuinely”. This brought hope and safety (“you feel safe”), likening this to a higher power (“support angel”).

It would seem the very thing the person with SE-AN feared, that is, relationships with others, could become the source of healing when trust and safety were co-created. Challenging the perceived safety of refuge with AN.

For some people the pathway to recovery opened through restoration of hope that recovery was possible. This was seen to occur through the support of therapeutic relationships and novel innovations with radically different approaches where “something new gives you a little hope” Dalton et al. p.240 [45].

##### Extract 24

*Then I found myself in a situation where I didn’t eat food, only liquid supplements. So I admitted myself for a week [Self-admission program] just to get out of that supplement swamp and start eating regular meals again. […] That was really a good admission, probably my first sound admission where I’ve felt like I was actually ‘on board’ myself. I’ve been treated against my will a lot, but this time I really set a goal, totally focused on it and used this week to get back to regular meals again. don’t need to be there for eleven months—if I just ask for it in time, it can be eleven days instead. […] If you just sacrifice two weeks, you gain ten months of freedom. It’s a pretty big thing* Strand et al. p 402

#### Sub-theme 4b: re-integrating self

From engagement in relationship and hope, meaningful, sustained change began, through the gradual emergence of a temporal yet integrated whole of self. Forever changed by the relationship with AN. Consistent with participants’ metaphorical expression the re-integration of self can be likened to Kintsugi – the Japanese Zen practice of repairing broken vessels with gold as ‘golden seams’. This metaphor is informed by these participant extracts:

##### Extracts 25

*formulating things in that way really helped me to start to put the pieces together a bit more. Putting the pieces together in my own way (Joyce et al p 2077 *[52]* )*

*Everything is still fragmented; things are still in boxes but its slowly coming together so I feel more Helen that I did a few months ago… I know who Helen is but where does the anorexia and the negativity fit? I know it’s there but where does it fit in to make the whole person? (Ross and Green p115 *[65]*)*

*...when you’re well or when it’s gone, you’ll just be you, you won’t be anorexic, you’ll be you (Williams et al. p 222 *[70]*)*

Kintsugi, is seen as a symbolic honouring of being broken (“fragmented”) as part of life’s path and finding growth and meaning. This involves a person finding value in the individual fragments of themselves to make ‘the whole person’, (“slowly coming together”). It also represents cognizance of their vulnerability—“I’m recovered 20 years but I know […] when I’m going through a bad time I could easily turn back to anorexia […] because I know it works” Foye et al. 2019 p 330 [50]).

Through a dis-entangling process of discerning what is me (“who Helen is”) and reconciling the anorexia and negative attributes (“where does the anorexia and negativity fit in”). This was built through reconnecting with themselves and building strength of self (“assertive enough”) to allow first for their own needs to emerge and then unashamedly honouring those needs. Even if this was perceived at the cost of honouring those of other;

##### Extract 26

*“…feel assertive enough to meet their [my] own needs and feel entitled to meet [my] own needs and accept responsibility for doing that, rather than meet someone else’s needs” (Blackburn p 435 *[42]*)*

To reach this phase, participants needed to acquire wisdom and insight about their AN relationship, as well as experience reparative interpersonal relationships and self-compassion. Understanding and making meaning of AN in their life as well as gaining perspective with time (“just before my 47^th^ birthday”)*(*McCallum and Allagia, 2021 g p 624 [58]) and discovering their own inner worth (“you deserve to belong here on earth for whatever reason”) *(*Patching and Lawler, 2009. p 18–19), took a long time*.* It evolved by feeling valued and accepted by others such that a kinder, more accepting relationship with self (“I am my best friend”) (Thoresen p 190 [68]) and less critical attitude towards self (“I fully unconditionally accept myself as I am”) could emerge, by first experiencing themselves as valued and worthy, in the eyes of others.

Sometimes the insights required for the reintegration of self, occurred many years after enlisting the functional adaptation, and onset of symptoms;

##### Extract 27

*In my forties, at a time in my life when I was relaxed and happy, I started to have flashbacks and I realized that I had been raped as a young child. I finally realized what had happened and I had a reason for the anorexia. With this understanding came some closure and I could get on with my life now […] this revelation was very distressing and intense for me. My need for control made a lot more sense. It seemed clearer to me why I wanted to torture myself and why I felt so controlled and angry as a child. (Dawson et al p 500-502 *[46]*)*

### Visual expression of the synthesis—greater than the sum of its parts

The overall finding in this meta-synthesis is that SE-AN, from a treatment seeking perspective, is conceptualised as a relational, existential disorder of the self. A visual expression of the synthesis, [37], anchoring the ensuing discussion of themes is shown in Fig. 3: *Conceptualising SE-AN: Recursive process of existential self in relation to other.* One participant’s metaphor (spider and its web) is woven into the visual expression to depict the extent by which SE-AN becomes a ‘trap’ and contributes to a disappearing self. This person-centered conceptualisation of SE-AN, synthesised across the extracted qualitative studies, encompasses phenomenology, treatment and recovery experiences.

This conceptual (see Fig. 3) demonstrates a recursive of existential self in relation to other (AN, people, food and life) and temporality of change/recovery through intra and inter-psychic (dis)connections and maintenance processes. The fractured self (1a Vulnerable sense of self) is organised and held together by an external agent (AN), using the participant metaphor of the spider. AN offers a pseudo sense of wholeness and is functional, thus protecting the person from their existential reality of isolation (2a Meaning of AN to Self). The AN then dominates (2b The Disappearing Self) and risks overtaking/ controlling the person and becoming self-maintaining. In [3] Global Impoverishment, the self becomes a shell, ghost, invisible, voiceless, or alien. Though this review, connection with “other” (4a opening the Self to Other(s)), built upon a foundation of trust challenges the deeply held fear of relatedness and healthy dependency in those with SE-AN. This affords a person opportunity to experiment with giving up the AN defense and potentially reach integration of parts of self (4b Reintegrating self) by being seen and held in trusting relationships and gradually nourished by the inter-personal, life and food. The re-integration involves a gradual, iterative process, enhanced over time, tethered by connection with ‘other’ and the wisdom, knowledge, and awareness of self, such that all parts of self can be seen more compassionately and therefore integrated. The ‘’kintsugi” or golden seams metaphor, borrowed from Japanese Zen practice, is in response to other metaphors shared in the participants’ dialogue (see extracts 25*)*. In this context, the seams represent fault lines and reminders of the past and can also be a richness of experience, tracing the evolving self;

## Discussion

This paper utilises meta-ethnography [37] to synthesise the findings of 36 qualitative papers, encompassing some 382 voices to propose an alternative, phenomenological conceptualisation of AN, from the perspective of those with a longstanding illness experience. Through participant engagement in treatment and sharing of those experiences, including to recovery for some, insights are synthesised to further understand how care might be improved for those in the processes of relinquishing a life from AN.

### Discussion of themes / thematic map

The first meta-theme of *Vulnerable sense of self* is consistent with current understanding of the development and maintenance of AN [82] as a complex interplay of personal, interpersonal, family, sociocultural, biological, and cognitive-behavioural aspects. Rationale to reconceptualise AN psychopathology, beyond predominant medicalized parameters and body image disturbance have emerged [83–86]. One of the intra-personal elements that has gained less attention, highlighted in this review is the notion of shame. This may contribute to aetiology in SE-AN and partly explain low treatment engagement and high drop out. There is potential for further re-shaming in the process of treatment and ego-syntonicity of AN in its compatibility with a self-view of worthlessness.

Shame is a silencing, controlling and stifling emotion, with its roots in formative development [87], such as interpersonal trauma and is exemplified by participants making sense of the origins of their pervasive unworthiness. The foundation of shamed self leaves a person compromised and unable to function from a position of wholeness and strength (*phase 1—vulnerable sense of self*), risking them becoming distrusting and estranged from others [76]. In other words, preventing them from building a robust place within self, able to seek nourishment from ‘other’. One aspect being food, but more broadly the person starves themselves of ‘life’ and other people, including therapeutic relationships. Developmental trauma, a potential source of fracturing of the self (shame) for some, gives rise to the need to adapt such as through disconnection from self, (in the extreme of this continuum via dissociative process [80]) and others, in order to survive.

Shame has been subject of a recent ED meta-analysis [88] and systematic review [89], suggesting that early changes in shame were associated with quicker reduction in ED symptoms [90]. Shame was found to be significantly associated with a medium to large effect size with all types of eating disorders, however illness duration was omitted as a moderator. This supports the notion that shame is another potentially fundamental consideration in the aetiological and theoretical conceptualisation and treatment for AN, which research has tended to overlook to date.

The negotiation of therapeutic relationships alongside a person with SE-AN is complicated. Engagement is a risk for a person, who has a blue print of mistrust (*vulnerable sense of self*), whereby even “the experience of connectedness in therapy may activate traumatic experiences of abandonment*”* Blackburn pg 438 [42]. Prematurely challenging this defence, or bursting “the bubble” [73] p 6, may be problematic for a number of reasons. For example, rupturing an already vulnerable capacity to build trust and connection with others could reinforce resistance and expose and re-shame the self. The person with AN who perceives themselves ‘defective’ underneath their carefully curated exterior of perfection, risks further rejection in exposing their shamed self. This vulnerability may partly explain the prolific accounts of aversive treatment experiences in this review. For example, re-experiencing relational trauma while seeking treatment through perceived controlling, punitive, dismissive or stigmatising treatment experiences, and where change and healing occurs through meeting a person in their ambivalence, and not confrontation or coercion ( [77] p123). Thus, relating in SE-AN is fraught, where a *vulnerable sense of self* makes it more difficult for people to engage in life and treatment relationships and possibly more likely that relational ruptures can occur.

In phase 2 *(meaning of AN to self*) this is potentially a critical point where a person risks illness entrenchment if treatment is not tailored to their needs. That is, treatments that offer maximal choice and autonomy within person-centred care paradigm versus involuntary, forced or coercive treatment. The latter disempowers a person to prematurely give up their *functional adaptation* in AN, beyond their level of self-support. This denies them the opportunity for growth on their terms [91], where unhelpful and unrealistic treatment experiences may risk hope and increase their defences. A complication for this hypothesis is the physiological consequence of starvation syndrome, unique to EDs, which can also be self-perpetuating. This can exacerbate other maintaining factors such as cognitive distortions and rigidity, leading to entrenchment and possibly, premature death. Finding the delicate balance between risk of illness entrenchment by prolonged starvation, versus activating defences further through coercive treatment is a clinical dilemma. The latter may progress AN, per the proposed phasic model (Fig. 3), towards a *disappearing self*. The interaction of unmet treatment needs in progressing AN to a dominant and enduring form requires further study.

It emerges as therefore critical to examine the ongoing place of AN in a person’s life and at their pace with an insight and awareness approach. That is, supporting a person to critically evaluate for themselves whether AN accelerates or slows down individual aspirations. That is, does AN alienate, enslave or liberate them [76]. If the person remains with their functional adaptation for survival (AN), the *Anorexia Nervosa Trap* (Figs. 2 & 3) is woven, whereby the AN takes a life of its own. Ironically consuming the entrapped person who is metaphorically and physically engulfed, until they ultimately cease to exist. A fixed ‘character structure’ emerges from the functional adaptation and ultimately the person and AN are fused.

Reclaiming self can become increasingly difficult as the person enters *global impoverishment* of the recursive process (Fig. 3). This pathway or trajectory illustrates the outcomes of AN by stage – whereby if the physical consequences of starvation don’t claim a person’s life first their capacity to exit out of relationship with AN may reduce overtime by virtue of the maintaining factors that develop consequently. Instead, life becomes a “prison”, “cage”, “trap” and death becomes the progressive loss of self. It is at this point that some people perceive suicide to be their only escape (Extracts 12 – theme 2a The Disappearing Self).

The gain of “being seen as a whole person” in the safety and containment of therapeutic relationships [92] or finding alternative treatment that connects a person with alienated aspects of themselves, can build hope and scope for the repair and rebuilding of the self by reclaiming control. Thus, *opening the self to others*, affirms that finding a healthy relationship with ‘self’ and healthy dependence on others are crucial aspects of the human condition, of being sustained, which go amiss for people with AN [93].

As further caution to respecting the experiencing person’s pace for change and engagement on their own terms, the insights required for *reintegration of self*, may not be immediately available to a person. These can only emerge as and when a person can assimilate such insights. That is, the acquisition of wisdom to see AN for what it truly is – a flawed, acontextual and harmful defence, that was once the only way they knew how to ‘survive’. This helped some participants to build a more compassionate relationship with themselves such that reintegration of self was possible through inter-psychic, temporal processes of change and recovery. This process of *reintegration of self* takes time, and is consistent with Duncan and colleagues [23] synthesis, who term this process “self-reconciliation” as a construct for AN recovery.

The finding in this meta-synthesis about the role of punitive treatment in illness persistence raises questions. If treatment focusses too much on the physical symptoms (i.e. BMI) it overlooks AN as a *creative and functional adaptation* to survival, required at a point in a person’s life, per phase 2 – *meaning of AN to self* and may risk entrenchment If the functional defence (restriction) is removed through punitive, rushed and or one-size fits all treatments, before a person builds resources and wisdom to appreciate its place in their survival, this may be negatively re-enforcing. Thus, undermining the person’s self-efficacy. Treatments such as dialectic behaviour therapy and Gestalt Therapy have not been widely used for SE-AN although the validation of the role of symptoms and behaviours are consistent with stances of such acknowledgement.

Intrinsic to all the themes in this review is the notion of *relationship* and the associated shifts towards connection versus disconnection and nourishment versus depletion and their interaction. That is, a person’s vulnerable relationship to themselves (“the self”) as informing their mistrusting relationship to others and difficulties building trust and then the loss of relationships through the course of the conflictual relationship with AN. There exists a dynamic and shifting process of engagement in these relationships throughout the course of illness, which can be underpinned or undermined by a foundation of trust in self, others and in life. It emerges as pertinent in this review that trust is the foundation of change and anything that risks distrust, (such as treatment and treatment relationships that rupture trust), reaffirms a person’s blueprint and risks causing further harm and illness entrenchment.

The voices of the experiencing person across these studies contribute to evidence of a complex and insidious condition, which so far largely eludes treatment efforts [18]. The picture emerges of a relational illness, whereby self is experienced as fundamentally flawed and undeserving of the nourishment available in the field or environment, and interpersonal contact to physical nourishment of food is restricted. The conceptualisation of phases of the illness (see Fig. 3) affords an appreciation of what we are asking of patients at various stages e.g., *disappearing self* and appreciation that AN is self-maintaining (Global impoverishment). As progression occurs, odds of ‘full recovery’, such that it is defined, may lessen. It is therefore imperative to consider what recovery means to the individual.

### Clinical applications and considerations

It is clear in this synthesis, that people with SE-AN can articulate both the phenomena of SE-AN as it is experienced, as well as their needs and preferences for SE-AN treatment to best support ‘recovery’. A practical translation, drawn from the composite papers for this review (see Table 4) highlight the wisdom, profound insights, and deep desire of people with SE-AN to contribute to the betterment of treatment. A commitment by people with SE-AN offering hope to all and calling the field to ‘do better’.

**Table 4:** Treatment needs from the wisdom of SE-AN experience

Therapeutic Stance
The therapist sees the person:
▪ as a unique individual
▪ outside the illness
▪ without preconceptions of how AN is for them
▪ struggling to find value in themselves and their life
**Therapeutic Relationship**
▪ allows time to build trust
▪ recognises relational struggles
▪ instils trust in self-efficacy, helps me to trust myself
▪ accepts me for who I am, especially the bits I ‘disown’ and see as unacceptable
▪ provides security with a boundary of treatment non-negotiables that prioritise my safety, and includes me in the process
▪ recognises that food may be a literal manifestation of being starved, a red herring
**Treatment**
▪ Helps me to understand my illness before I am expected to give it up
▪ Recognises that my illness really works for me and is the best way I know how to survive^a^
▪ Recognises my illness is compatible with the low value I place on myself^a^
▪ Helps me to build meaning about what AN is in my life
▪ Allows space in treatment, to address my unique concerns beyond the treatment manual
▪ offers me choices and maximises my autonomy so I can build my own self-efficacy
▪ Measures me not by my weight
▪ Allows me the freedom to create the terms for my life
▪ Helps me to connect to all aspects of my being and offers a variety of treatment adjuncts
▪ If I need to go to hospital, offers me emotional support as well. Recognising when I am terrified and that I may have past trauma from previous admissions too
▪ **Recognises the broader context of living with SE-AN, including;**
- Judgement and misunderstanding of my illness
- Being enslaved to AN, it is the ‘master’
- Appreciation of my profound losses to SE-AN
- Helps me to make peace with my life I have made alongside AN
- Offers me the care that I need in line with my stage of my illness. I’m not an adolescent

^a^recognising the broader aspects of ego-syntonicity beyond control of body image and weight

Treatments for AN are agnostic with regards to cause. This review positions AN as an existential crisis of the self which may lend to exploratory psychotherapy to support the process of individuation and re-connection to the emergent self at an earlier stage of illness. It is undetermined if addressing aetiological processes earlier in AN treatment would reduce its persistence. At the later stages of global impoverishment, it is unknown if a person, so impaired by their AN can meaningfully engage in deep psychotherapeutic work of asking questions about their own existence. This remains untested and conventional wisdom is that re-feeding precedes psychotherapy. From the perspectives of those who experience SE-AN, engaging in treatment and recovery is perceived to be a risk. For example, a person with an already *vulnerable sense of self*, to confront their emotional pain of a life half lived, with a body half left (*disappearing self*) then so *globally impoverished* to AN, may not be accessible. The perceived treatment inadequacies articulated by participants may implicate them in the role of illness persistence and this also warrants further testing. The alternative position, where therapy is slow paced, individualised, relationally orientated and insight and awareness based, with reduced focus on weight-restoration have not been tested. Specialist supportive clinical management (SSCM), may offer a first step to defining such a treatment for SE-AN within proposed understandings of its efficacy [94], with the potential to encompass attributes described by participants in Table 4.

A recent treatment innovation Maudsley Model for Anorexia Nervosa Treatment for adults (MANTRA) has been founded on a comprehensive theoretical basis of a cognitive-interpersonal model of maintenance [95]. This model acknowledges the “valued nature of AN” as consisting of “pro-anorexia beliefs” in the mid-phase of therapy, (chapter 5 p 84). AN as an identity is also the focus of the penultimate chapter of the treatment manual (p 191). The profound unworthiness or shame processes identified in this review, are not explicitly targeted in the modules. Given the complexity of addressing shame in therapeutic interventions [96] how this might be integrated into a manualised treatment is incomplete. MANTRA treatment is tailored to the needs of the individual, with chapters to be determined collaboratively with patient, and delivered via a motivational stance. It has been argued that attempting to target too many factors in AN concurrently is problematic [97] and insufficient attention is paid to the importance of the therapeutic relationship in manualised therapies suggesting the ‘how’ may be even more important than the content for people with SE-AN. These factors may require additional consideration in treatment transferability to SE-AN [98] p 188.

The concept of treatment as help versus harm is considered a ‘cross cutting’ theme, as people sought treatment throughout the various stages of illness and treatment traversed and impacted all themes. For many people, treatment did not meet their needs and preferences which emerged in all treatment experience papers and cited in papers of other foci e.g., phenomenology, totalling 15 papers. At worst, treatment was cited as a source of harm (e.g., Broomfield et al. 2021). Treatment was perceived by some as reductionistic, where the focus was restricted to ‘symptom reduction’ (weight) which could be experienced as abandoning, rejecting, discriminatory and, causing harm. This focus reinforced the need to be under-weight to have the need for help validated and prevent perceived abandonment. The low regard for aetiology and functionality in treatment gave participants the sense that those aspects are unwelcome and at worst, increased their suffering*,* potentially risking hope*.* The stance experienced by participants of reductionistic treatment is inconsistent with conceptualisations of AN recovery articulated elsewhere [23]. Thus, as a clinical implication, seeing the person within the full context of their illness with attention to individual aetiology arises as important. Tailoring treatment to the individual may reduce barriers to treatment engagement by not threatening perceived useful symptoms (food and body practices) with potential for paradoxical outcomes for ambivalence and change and building self-efficacy. Where people felt a sense of agency in their treatment, through for example, a self-admission program this led to empowerment [99] p 1691. This was also true for intensive community treatment and peer support, in which former was perceived to be more closely aligned with ‘real life” and supportive of risk taking and change where people can experiment with new behaviours at home, grounded by supportive others. Peer support was cited to reduced barriers, by offering a safe space with less judgement and was felt as less threatening and more empowering. These factors would benefit from further investigation and translation to clinical practice.

Of note, music therapy and yoga had the potential to connect people to alienated aspects of themselves, namely their bodies and spirituality. Music created by a person was experienced as freeing and enabled a deeper connection to their internal world. This connection with self and feelings was also fostered during yoga and is consistent with other theoretical literature about building connection with the emotional or felt-sense of self [100, 101] as the precursor to forming an identity beyond AN and towards recovery [84]. Overall, peer support programs [72], self-admission [74], community support [51] as well as radically different treatment approaches such as experimental rTMS [45], music and yoga therapy (encompassing spiritualism and self-compassion) built upon a person’s connection with themselves, others and fostered hope. When considering the global impoverishment people experience to SE-AN, and the significant functional impairment described, the role of social work and occupational therapy support for people with SE-AN requires further exploration [102].

With a full appreciation of the life-worlds amplified through the collective voices in this review, it emerges as essential that care is adjusted in line with the reality of life for each person. Essential for SE-AN, that the full context of the experiencing person is considered, and care is shifted to align with what is possible and accessible to them, rather than pre-determined expectations that risk exacerbating hopelessness and causing harm [103]. That is, working alongside people to define their individual place of recovery [104], suspending expectation of what we consider an acceptable life [105] and supporting them to find their own personal agency and unique recipe towards wellness. To be truly accepting, in the hope that a person has the best chance to embrace and accept their authentic self by being seen in therapeutic relationships [92].

Other areas of mental health care label this as recovery orientated care [see Kiely, Hay and Robinson, 2023 In Press] which within contemporary understanding involves building your best life alongside an illness where complete cure may not be possible. This is not to discount the possibility of cure, as defined by the experiencing person. Paradoxically, by adjusting our goals for treatment to better meet a person where they are, has been shown to have improved outcomes with better engagement and retention in treatment and improved quality of life for that which remains (Touyz et al. 2013 [106]). Essential to maintaining *hope* is an appreciation that change is a temporal, recursive process that when dealing with a complex existential illness as demonstrated in this review, the ‘tipping point’ often referred to, is a gradual, iterative process. That which requires time, space, safety and all of the attributes eloquently articulated by those with SE-AN in this review (see Table 4), where the light bulb moment is rather a series of awakenings in the direction of change.

Hope versus hopelessness, named as a cross-cutting theme, alongside the temporal processes. Hopelessness was built upon repeated, failed treatment attempts, feeling neglected as a patient group, treatments that did not meet needs and preferences, and most notably harmful ruptures to the therapeutic relationship from loss of hope from others. This is a pivotal point where if the treatment mirrors the illness itself – rigid, rejecting, conditional, superficial, discriminatory, dehumanising- then the effects are reported as traumatic and harmful and contribute to hopelessness and further entrapment to AN.

The treatment attributes people with SE-AN have described (Table 4), can support finding hope, personal capacity for development and growth, and respect a person’s ambivalence. Conversely, when absent from treatment, a person is potentially left with hopelessness, defeat, and repeated treatment failure. Reinforcing the shamed view of self, promotes the fatal refuge in AN that can be perceived as more tolerable than treatment or a life without AN.

## Limitations and strengths

To our knowledge, this paper is the first meta-synthesis of SE-AN with 36 items of scholarship, encompassing grey literature, and a resultant two unpublished theses informing the analysis. Rigorous methodology, in which 78 authors were contacted for further information to determine inclusion further expanded the sample. There were potentially additional papers that may have met the inclusion criteria (being the sum of authors who either declined to participate, information was unavailable or who were uncontactable), and papers published after July 2022. However, 36 papers is considered a large synthesis and all authors agreed that saturation was reached [107]. As a further methodological strength, a proportion of consensus rating was achieved with more than one author for the inclusion of papers and quality allocation.

The sample as defined in Table 2 includes 8% of people who identify as male gender which is comparable to community prevalence of EDs in men and some people identified as homosexual. None of the respondents identified as non-binary or transgender, which represents a limitation given emerging literature about higher prevalence of non-binary and transgender as manifest in AN (for SE-AN unknown). The predominance of ‘treatment seekers’ (> 86%) may not reflect the broader experiences of a community sample and homogenisation to a predominantly Caucasian sample overlooks cultural representation.

There is triple hermeneutic inherent in meta-ethnography, where three layers of subjective abstraction occur. That is, the person making sense of their world, the researcher making sense of the person making sense of their world, and finally the synthesiser making sense of the two. To mitigate risk of bias, three authors were involved in the construction of the meta-themes and cross cutting themes, representing a strength of the synthesis.

True to all qualitative systematic reviews, findings should not be interpreted as a solution or diagnostic answer but seen as a representation of a set of perspectives. Also noting that a high risk of bias was assigned to some studies (see Table 1). Imposing a theory or conceptualisation onto a person (i.e., fitting a person to a formulation) was strongly opposed by the voices of AN experience and implicated in illness persistence, so it would be ironic to transpose a conceptualisation proposed in this paper to other people’s experiences. As a further limitation, it was not possible to member check the conceptualisations (Figs. 2 & 3) generated from the review, with participants of the primary studies.

The type of psychotherapeutic work proposed in this paper is ideally long term and demanding of both patient and therapist and workforce issues, access and tenability are limitations to be considered. The demands of the therapist are immense, including the need to confront personal biases, idealisms and preconceptions and persist long term with a patient through the inevitable ups, downs, and relapses of life with SE-AN, as well as the ability to tolerate the profound grief and loss that may emerge as part of the shared encounter. It is unlikely whether current tertiary training equips mental health providers to offer the type of support that may be needed for this pathway and further post-graduate study indicated. One size does not fit all with regards to diagnosis, treatment, or ways of being or living and a person-centred paradigm is our first leap towards meeting an individual in their complex relationship to themselves, others, life and AN.

### Future research directions

True to the ethnographic approach, care has been taken to maintain fidelity to the original authors themes. When reading all papers ‘en-masse’ it became apparent that additional themes were present which had not explicitly been identified as such, within the papers. These included the phenomena of dissociation, trauma, and metaphor. A starting point of the verbatim quotes (data) pooled, rather than working from primary authors themes could allow for more objective, fresh interpretations of collective data and could be considered for any future SE-AN meta-synthesis.

Comparatively fewer studies (*n* = 4) related specifically to recovery and while references to recovery were found within other papers, this may indicate that the possibility of recovery is lower in a more enduring state of illness and current definitions of recovery are limiting. As such, qualitative accounts of SE-AN recovery, should be published as a priority to build a more comprehensive picture of how people may (or may not) negotiate a life alongside SE-AN. Furthermore, emphasising that in the absence of a definition for recovery [108], this is best defined by the experiencing individual and what it is a person wishes to ‘recover’ from their life. There is an absence in the literature of research that distinguishes duration of the illness and impact at different timepoints and identifying features that may be predictors of enduring illness as such, high quality longitudinal mixed-method studies are needed.

The results of this review suggest that further investigation is needed into; (1) the putative role of the experience of shame in the aetiology and conceptualisations of AN and (i) as a barrier to treatment seeking (ii) how shame processes might be integrated into a manualised treatments (2) Adaptation and testing of treatments according to a phasic model for SE-AN, including capacity for meaningful engagement in psychotherapeutic work for those in phase 3 (global impoverishment) (3) the significance of the therapeutic relationship for people with SE-AN, described as a relational illness where mistrust is manifest (4) the impact of treatments aimed at connecting a person with alienated aspects of themselves in SE-AN as described in this review i.e. self-admission programs, yoga, music therapy, peer support (5) the role of putative factors in AN persistence such as treatment inadequacy, trauma (especially interpersonal trauma) and potentially dissociative processes (6) the role of self-compassion in recovery (7) the efficacy and ethics of addressing aetiological processes earlier in AN treatment (alongside re-feeding) (8) the role of perceived threat of premature loss of coping mechanism as a barrier to treatment seeking (ED behaviours and finally (9) the efficacy of relational, non-manualised therapies e.g. Gestalt, narrative and existential approaches.

It emerges as pertinent that the hypothesis of ‘harm in treatment’ or ‘treatment trauma’ and its role in illness persistence is urgently subject to further testing. That is a) accounts of trauma as part of treatment experiences and b) investigating the feasibility and efficacy of implementing treatments in the first instance that, prioritizing a person’s physical safety, meet a person at their point of readiness, and work alongside their existential defense for survival.

Has the time come to confront limitations in the dominant psychology paradigms and trial different ways of working with a complex and insidious illness? It is possible that true treatment innovation may be found by *deepening* of clinical work to a greater level of sophistication, both individualised and expansive, engaging all aspects of being human. It is incumbent on researchers, clinicians, and communities to collaborate in testing the hypotheses proposed by the collective voices within this paper. The Specialist Psychotherapy with Emotion Kent and Sussex (SPEAKS) feasibility trial [109] has a theoretical basis of strengthening the emotional self through integrative emotion focused therapy and dialogically based, schema ‘self-parts” therapy. Results of this trial may be a first step toward understanding the role of these neglected aspects of treatment. The inclusion of criterion for SE-AN related to the ‘global impoverishment of self’, as identified in this review, warrants consideration within future testing of putative defining features. The validated IDEA (IDentity and EAting disorders) instrument [110], has potential to assess the ‘global impoverishment of self’, being the psychopathology of embodied disturbances of identity in SE-AN.

## Conclusion

This systematic review offers an alternative conceptualisation of SE-AN developed from the many voices of lived experience across the included studies. SE-AN is positioned as a successful defence or *functional adaptation*, serving varied functions for people, such that a person with a *vulnerable sense of self*, complicated by shame processes, is protected from the pain of their perceived inadequacies. A person’s relationship with AN can become all consuming, fuelled by starvation to the exclusion of all else such that a person disappears. A conflictual AN relationship ensues, with potential to take on a life of its own. Ultimately AN controls the person, who ironically enlisted it to help feel some semblance of control in an internal and external world that feels intolerably messy and overwhelming. This paper has found that the therapeutic role of treatment for SE-AN is mixed at best, but that with the right ingredients, as defined by the experiencing person, relinquishing life dominated by AN is possible, building a picture of hope for all [111]. This meta-synthesis thus presents a process of change and recovery. Change that is possibly a slow, iterative process built on the foundation of connection with self, others and life. Future areas for research, as well as clinical implications have arisen from this review. The voices of experience, synthesised and translated for the purposes of this meta-analysis, propose an alternative picture of SE-AN. This poses a challenge to current conceptualisations of AN and calls for treatments to engage with the complex intra and inter psychic processes of the SE-AN, more fully. The ‘global impoverishment of self’, found in this synthesis of AN experiences, should inform proposed diagnostic criteria for SE-AN.

### Supplementary Information

**Additional file 1.** Search Terms.

**Additional file 2.** Box 1 Summary of themes: all literature.

## Data Availability

The datasets used and/or analysed during the current study are available from the corresponding author on reasonable request. All data is included in the original papers, as referenced.
