# Supplementary material for: Conceptualisation of severe and enduring anorexia nervosa: a qualitative meta-synthesis
Source: BMC Psychiatry. 2023 Aug 18;23:606. doi: 10.1186/s12888-023-05098-9 (PMC10439651; doi:10.1186/s12888-023-05098-9)
Supplement: Supplementary file 1 — Additional file 1. Search Terms. [file 12888_2023_5098_MOESM1_ESM.docx]

**Additional File 1 Search Terms**

Table 1: Database search terms *psychINFO & CINAHL (Ebscohost), SCOPUS, MEDLINE & EMBASE (Ovid), Scopus and Google scholar*

| **Population** | **Concept** | **Context** |
| --- | --- | --- |
| Anorexia Nervosa (long duration) | Experiences, Treatment, Phenomenology, Recovery | Qualitative research |
| Anorexia Nervosa | Adaptation,Psychological  adjustment  experience  growth or adapt*  model psychological  posttraumatic growth  psychological  psychothera*  Psychotherapy  recovery or resilience  resilience psychological  theory psychological  therap*  Therapeutics  Therapy  treatment*  treatment experience  well-being psychological | Behavioral Research  case report  Case Studies  ethno*  Ethnographic Research  Ethnological Research  Grounded Theory  hermeneutic*  interview  interview as topic  narrat*  Narration  personal narrative  phenom*  Phenomenological Research  Phenomenology  qualitativ*  Qualitative Studies  questionaire*  Semi-Structured Interview  surv*  Surveys  surveys and questionnaires  verbal communication |
