# Supplementary material for: Conceptualisation of severe and enduring anorexia nervosa: a qualitative meta-synthesis
Source: BMC Psychiatry. 2023 Aug 18;23:606. doi: 10.1186/s12888-023-05098-9 (PMC10439651; doi:10.1186/s12888-023-05098-9)
Supplement: Supplementary file 2 — Additional file 2. Box 1 Summary of themes: all literature. [file 12888_2023_5098_MOESM2_ESM.docx]

**Box 1 Summary of themes: all literature**

| UNREADY / UNABLE TO CHANGE | improved relationship with food | | |  |
| --- | --- | --- | --- | --- |
| external locus of control | less tired |  |  |  |
| internalised AN | One step forward, two steps back | | |  |
| perceived treatment as unhelpful | guilt and shame versus recovery and success | | |  |
| little understanding of illness | AN paradox |  |  |  |
| unawareness of cause and function of AN | POSTPARTUM |  |  |  |
| Low motivation | self / identity |  |  |  |
| TIPPING POINT OF CHANGE | relationship to self, other(s), AN | | |  |
| worn out by AN | putting the pieces together in my own way | | |  |
| understood by others | At the crossroads between remission/ relapse | | | |
| increase in insight | prominence of fragility when choosing a partner | | | |
| AN externalized | An asymmetric commitment | |  |  |
| internal locus of control | When anorexia reveals itself | |  |  |
| ACTIVE PURSUIT OF RECOVERY | compromised intimacy | |  |  |
| recovery-orientated skills utilized | Menage a trios |  |  |  |
| active pursuit of symptom reduction | from active to passive distancing | | |  |
| persistence and determination used | A petrified system | |  |  |
| increase in self-efficacy | interpersonal avoidance | |  |  |
| REFLECTION / REHABILITATION | intrapersonal avoidance | |  |  |
| maintaining changes developed | control / restraint | |  |  |
| reflection | achievement / purpose | |  |  |
| self-acceptance | self-punishment / denial | |  |  |
| COMPLEXITIES OF MIDLIFE | NEGATIVE ASPECTS | |  |  |
| changing roles and responsibilities | *interpersonal avoidance* | |  |  |
| protection of professional identity | *impact on family* | |  |  |
| FEELING DISMISSED / MISUNDERSTOOD BY HEALTHCARE | *achievement / purpose* | |  |  |
| INCREASED STIGMA | *physical health / appearance* | |  |  |
| internalised stigma | All on our own | |  |  |
| social stigma | becoming needless | |  |  |
| feeling lesser than | Transition |  |  |  |
| PATHWAY TO RESILLIENCE: wisdom gathered over a life half-lived | Trauma |  |  |  |
| capacity to reassess | interpersonal, body trauma / diet food trauma | | | |
| realisation that life is time-limited as a turning point | functionality |  |  |  |
| freedom to live their life | Identity |  |  |  |
| self-discovery | a shifting process throughout the illness trajectory | | | |
| resilience after adversity | grief / letting go | |  |  |
| Control | Failure of current models of treatment | | |  |
| controlled by the condition | The eating disorder had a negative impact on interpersonal relationships | | | |
| relinquishing control | Lack of flexibility in social situations | | |  |
| regaining control | intense experience of negative feelings | | |  |
| connectedness | lack of social skills | |  |  |
| a sense of not belonging | Isolation due to fear of food or body image concerns | | | |
| ultimate disconnection | Fear of being judged for having an ED | | |  |
| reconnecting with life | Poor capacity to concentrate on conversations due to eating disorder thoughts | | | |
| conflict | Social difficulties ascribed to comorbid disorders | | | |
| self-acceptance | need to fit in |  |  |  |
| To become alive | Need to feel control | |  |  |
| more aware | Need to hide due to inadequacy or way to self-punish | | | |
| kinder toward herself | Need to distract from loneliness and isolation | | | |
| Dynamics during pregnancy | Restriction of energy intake as a mean of silencing negative thoughts and emotions | | | |
| *a new goal* | readiness of change | |  |  |
| *honey moon* | pregnancy as healing / purpose / locus of control | | | |
| *perceived positively by all, bodily pleasure and pride* | Isolation, Sex, Sexual Identity | |  |  |
| *mother-daughter positioning* | Masculinity |  |  |  |
| Understood by others | *masculinity through exercise and food restriction* | | | |
| *fears and AN relapse* | *masculinity and treatment* | |  |  |
| *redemption and pride* | Not working 9 to 5 | |  |  |
| Sadness | identity |  |  |  |
| avoiding awareness of sadness | avoidance |  |  |  |
| inhibiting expression of sadness | difference |  |  |  |
| Anger | control |  |  |  |
| avoiding anger | gratitude |  |  |  |
| inhibiting expression of anger | responsivity |  |  |  |
| fear | communicate |  |  |  |
| supressing | company |  |  |  |
| disgust | addiction / anxiolysis | |  |  |
| avoiding | anger / hate |  |  |  |
| Making sense of AN | health |  |  |  |
| interpersonal relationships | emotion avoidance | |  |  |
| subtheme general relationships | devaluation |  |  |  |
| relationship with staff / therapist | social impairment | |  |  |
| Battling with the anorexia | shame / takeover | |  |  |
| emotional regulation | fear / distress |  |  |  |
| ED as coping strategy | annihilation |  |  |  |
| avoidance of emotional stimulation (positive and negative) | betrayal / pretend | |  |  |
| emotional expression | loss / waste |  |  |  |
| voicelessness of having no emotional language | food obsession | |  |  |
| emotional awareness | others |  |  |  |
| dampened by ED and lost, frustrated and confused by emotional experiences and arousal | emptiness |  |  |  |
| emotional connections | physical - severe symptoms, minimization, self management | | | |
| seeking solace in the ED | PSYCHOLOGICAL | |  |  |
| function of AN | unworthiness |  |  |  |
| self criticism versus self acceptance | clinical frugality | |  |  |
| isolation versus connection | depression, OCD | |  |  |
| hopelessness versus hope | time-keeping |  |  |  |
| stuckness versus change | POSITIVE EXPERIENCES RELATED TO ED | | |  |
| paradoxical functions of exercise | social |  |  |  |
| diverging experiences of exercise | isolation, avoidance, no intimate relationships | | | |
| I feel like im being compelled to exercise - ambivalence /control | family |  |  |  |
| I should rather have trained : limiting the societal and relational world | food |  |  |  |
| limits social relationships and a source of conflict in relationships | obsessions |  |  |  |
| minimizing and rephrasing | relationship to illness | |  |  |
| weakened self | relationship to themselves | |  |  |
| struggling for control | relationship to others | |  |  |
| Controlled by the illness | Suffering but not in silence | |  |  |
| concealing | staying childlike | |  |  |
| the illness | being seen as a psychiatric patient | | |  |
| the self - shame and stigma | self-image /identity | |  |  |
| Feeling consumed | addictive behaviour | |  |  |
| Connective Tissue | innocence |  |  |  |
| relationships | Being understood | |  |  |
| maintaining SE-AN | understanding self | |  |  |
| contact and connections with others | connected in the here and now | | |  |
| anorexia as a conductor | connected to the past | |  |  |
| Best Friend, best enemy | connected to the future | |  |  |
| personification of anorexia | access to treatment | |  |  |
| blame attribution | treatment structure | |  |  |
| dependency upon services | therapy that focussed on food / weight | | |  |
| understanding the development of AN | Inpatient treatment that did not include therapy | | | |
| triggers | outpatient therapy that was short term | | |  |
| perfectionism | overly rigid CBT | |  |  |
| chronic low self-esteem and self-worth | treatment providers | |  |  |
| Experience AN as functional | being treated as a whole person and having real relationship | | | |
| distraction to cope | programme acceptability | |  |  |
| safety in predictability | she understands me and could relate to me | | |  |
| focus away from lack of valued roles | reconnecting with the world. Asking questions and being challenged | | | |
| Anorexia Identity | instilling hope - recovery is possible | | |  |
| negative effects of anorexia | function of anorexia | |  |  |
| negative impact on life | yoga experience | |  |  |
| lack of understanding by family / friends | Authenticity of the relationship | | |  |
| social life | the importance of safety | |  |  |
| negative impact on family/friends | externalization of the ED | |  |  |
| hopelessness | The power of hope and optimism | | |  |
| Shame | maternalism |  |  |  |
| Solitude | Experience of treatment / refeeding | | |  |
| Salvation | relationship with staff / therapist | | |  |
| Habitus and Embodied Routines | what did participants want or not want from treatment  staff pessimism in treatment of cAN | | |  |
| safety and routine | treatment experience | |  |  |
| Sociocultural elements which support and sustain peoples ED practice | seeking help reaffirms personal weakness | | |  |
| it’s the glue that holds everything together | Help! I need somebody - bedlam revisited | | |  |
| AN taking over self | all sought help with high latency and wait lists | | | |
| AN having a voice | poor health care experiences overall | | |  |
| AN protecting the self | positive working relationships | | |  |
| Sharing the self with AN | waiting lists for services | |  |  |
| Being no one without AN | cycle of accessing services | |  |  |
| Discovering the real me (accepting the fear) | feeling desperate for help | |  |  |
| function of anorexia | overwhelmed by food and weight gain | | |  |
| social stigma | transitions |  |  |  |
| feeling lesser than | negative experiences of staff attitudes | | |  |
| other patients | treated as a group of patients | |  |  |
| admission presupposes deterioration | neglect judged abandoned versus accepted and cared for | | | |
| perceived failure | experience of being with other patients | | |  |
| fear of being forced to stay longer | supports / friendships | |  |  |
| applicability | competitive / comparisons | |  |  |
| simultaneously requires and fosters motivation and maturity | impact of other distress | |  |  |
| lack of motivation | Feeling Dismissed misunderstood by health care | | | |
| requires encouragement | increased stigma | |  |  |
| risks becoming self-destructive | failure of current modes of treatment | | |  |
| risks impending independence | Treatment |  |  |  |
|  | within a strong therapeutic relationship  emotional awareness and understanding  emotional avoidance  emotional expression and negative beliefs  extreme emotional responses interactions and relationships  emotional awareness and understanding  emotional avoidance | | |  |
